# Being a ‘good’ doctor: Understanding and managing professional boundaries is challenging and can lead to stress and burnout

**DOI:** 10.1177/10398562231191662

**Published:** 2023-08-10

**Authors:** Lisa Lampe, Rita Hitching, Trent Ernest Hammond, Jeannie Park, Dominique Rich

**Affiliations:** School of Medicine and Public Health, 64834 University of Newcastle College of Health, Medicine and Wellbeing, New Lambton Heights, NSW, Australia; School of Medicine and Public Health, 64834 University of Newcastle College of Health, Medicine and Wellbeing, New Lambton Heights, NSW, Australia; Nepean Clinical School, 64834 Faculty of Medicine and Health, The University of Sydney, Penrith NSW 2751, Australia; 3960 Northern Sydney Local Health District, St Leonards, Australia; School of Medicine and Public Health, 64834 University of Newcastle College of Health, Medicine and Wellbeing, New Lambton Heights, NSW, Australia

**Keywords:** boundary challenge, boundary crossing, boundary violation, professional boundary, burnout, doctor, physician, medical student, wellbeing

## Abstract

**Objective:**

The aim is to increase the understanding of non-sexual boundary challenges and potential personal and professional impacts on doctors and medical students.

**Method:**

We examined peer-reviewed and grey literature and published commentary and cases from Australian health practitioner boards and medico-legal insurance companies. Key ideas relating to the objective of our study were subsequently framed into a narrative.

**Results:**

Compared to ‘sexual’ boundary crossings, the literature examining ‘non-sexual’ boundaries is scanty, fragmented, and difficult to find. There are gaps in knowledge around the prevalence and consequences of non-sexual boundary challenges and crossings, although the safety and wellbeing of health professionals and patients are of concern. Non-sexual boundary crossings may represent a ‘slippery slope’ to boundary violations. Opportunities for doctors and medical students to access relevant training appear limited.

**Conclusions:**

We identified several categories of boundary challenges based on context, the nature of the existing relationship, and the type of behaviour. Non-sexual boundary challenges may be related to stress, burnout, and risk for future boundary violations. Future research to investigate the impacts on doctors and medical students in maintaining professional boundaries in their relationships with patients and colleagues, their specific training needs, and the effectiveness of training in reducing work-related stress and burnout is needed.

Professional boundaries have been defined as the ‘edge’ of appropriate professional behaviour, maintaining the expected and accepted psychological and social distance between health professionals and patients.^1^ This distance protects patients from the power differential^2^ and helps preserve the relative objectivity of medical decision making.^3^ Boundaries also refer to the limits on acceptable interactions between the health professional and their patient. Interactions with the patient should arise from and be directed towards meeting their medical needs, be within the scope of the health professional’s expertise, and contextually appropriate. Acceptable boundaries can also be understood with reference to the health professional’s usual practices, including location of consulting and hours of practice.^4^

The doctor–patient relationship has long been regarded as fiduciary in nature, in that doctors are expected to put the patient’s needs before their own and avoid conflicts of interest.^5^ There is a wide agreement in the literature that, as the individual with greater power in the relationship, it is the health professional’s responsibility to establish and maintain appropriate boundaries.

## Aims

The main purpose of our literature review was to synthesise information about non-sexual boundaries and develop a new understanding of the topic to support planned research. We sought to understand more about types and impacts of non-sexual boundary challenges.

## Data sources and methodology

A broad search (peer-reviewed, professional, and grey literature) was conducted using the keywords above. The search was hampered by association of the term ‘professional boundaries’ with different meanings including interprofessional scope of practice or, as in the current review, professional relationships. Although the primary focus of the review was on doctors and medical students, there was a much larger literature derived from the nursing, psychology, and social work professions which was included where the research team considered the findings to have relevance for doctors and medical students. A search of the codes of conduct published by peak or certifying health professional associations in Australia (including medicine, dentistry, physiotherapy, occupational therapy, chiropractic, and pharmacy) was conducted. Finally, the websites of medical defence associations in Australia were searched for publications concerning professional boundaries. There were no exclusion criteria.

A qualitative approach based on thematic analysis was adopted. Categories of non-sexual boundary challenges were developed through an iterative process involving consideration of suggestions in the literature and interpretations of the literature by the research team. The literature on impacts of non-sexual boundary crossings was synthesised into a narrative.

## Results

### Categories of non-sexual boundary challenge

We developed 10 categories of non-sexual boundary challenges presenting the risk of boundary crossing or violation. Category names reflect common usage in the literature or descriptive names developed by the researchers. Table 1 lists categories developed together with brief descriptions and examples.

**Table 1.** Categories of non-sexual boundaries and potential impacts

Category name	Brief description or example	Potential impact/s
Clinical favour	Requests for medical services by non-patients. For example, ‘corridor consultations’; requests by a colleague for prescriptions or referrals	Harm to person seeking medical service because doctor may not know full medical history; discontinuity of care; loss of medical decision-making objectivity because interaction is occurring outside of usual doctor–patient relationship
Dual relationship	New or existing social relationships in addition to the doctor–patient relationship, for example, a doctor becomes a client of a patient who is a fitness trainer	Conflict of interest; deliberate limitation of scope of medical review to preserve patient privacy; power differential. Damage to both personal and professional relationships if things go wrong
Edge of clinical role	The limits of indicated medical care. From a Medical Defence Organisation, the example of a doctor who sent a patient music and picture files designed to be uplifting; as another example, offering a patient a lift in the car	Could create unrealistic expectations in the patient or damage to the doctor’s reputation if motivations are misconstrued (in the real-life case relatives feared the patient was being groomed for an inappropriate relationship and made a complaint)
Emotional guarding	Deliberately withholding expression of affect or responding to a patient’s display of emotion. For example, with a patient perceived as ‘too needy’	Psychological aspects of patient care compromised; objectivity may be adversely impacted
Gift-giving	Gifts offered by patients to doctors or vice versa	May create a sense of obligation that could impact objectivity of medical decision making; risk to reputation via impressions that may be created
Non-sexual physical contact	For example, hugging	May create false sense of intimacy and impact patient expectations; risk to reputation
Self-disclosure	Sharing personal information or experiences	May create false sense of intimacy for patient; risk to reputation
Social media	Becoming ‘friends’ on social media	May create false sense of intimacy for patient; risk to reputation and privacy
Special patient	Making special arrangements for a patient, such as seeing them outside of usual hours or in other than the usual practice setting	May create unrealistic expectation for continued special treatment, and patient may become angry or resentful if this doesn’t happen; could be the start of the ‘slippery slope’ to boundary violation; may create reputational risk if situation misconstrued by others; departure from usual treatment approach may lead to risks for patient
Transactional	Substitution of usual processes around payment for medical services with alternatives, such as bartering of goods or services. For example, a patient offers to provide an artwork or the doctor suggests that they could clean the office in return for medical services. Can also be seen in supervisor/student situations, for example, offering employment to the student outside the supervisory relationship	As the less powerful in the relationship, the patient may feel unable to refuse a request for alternative payment type or to negotiate the true value; fear that to decline may adversely impact treatment; risk to reputation if others form the view that patient (or student) is being exploited

### Impacts of boundary drift, crossings, and violations

In an influential and widely cited article, Gutheil and Gabbard^6^ described a psycho-therapeutic framework for understanding professional boundaries. This seminal article distinguished ‘boundary crossings’ from ‘boundary violations’. Since then, ‘boundary drift’ has been described,^2^ as contemplation of a potential boundary crossing, or a behaviour that is close to the boundary.

In a boundary crossing, the health professional engages in an interaction with a patient that is outside of indicated therapeutic interventions or the professional’s usual practice. Boundary drift and boundary crossings may not necessarily be intentional nor cause harm to the patient. However, harms may be subtle, including loss of objectivity, conflicts of interest, distorted patient expectations, or a perception of patient exploitation. They may increase the risk of future boundary violations (the ‘slippery slope’).^7^

Boundary violations, by accepted definition, cause or have the potential to cause harm to patients and involve a behaviour that prioritises the health professional’s wants or needs over the patient’s.^8^ In the medical profession, even the perception of a boundary crossing can harm a doctor’s reputation.^9^ In health settings, the focus on boundary violations and crossings has traditionally been on sexual transgressions. However, many non-sexual categories of boundaries are described in the literature, mainly according ‘special’ patient status, providing clinical favours to non-patients, dual and multiple relationships, accepting and receiving gifts, physical contact, self-disclosure, and social media interaction.

Various influences on boundary crossing behaviour have been described, including the health professional’s own emotional vulnerability, ‘moral weakness’, exploitative character traits, and ignorance.^4,10^

### Boundaries in different contexts, locations, and specialities

Some practice contexts may offer particular challenges to maintaining boundaries, including rural, remote, or isolated practice, where social relationships outside the professional one are common and often unavoidable, thus creating ‘dual’ or ‘multiple’ relationships with a patient.^11^ Other contexts include doctors in highly specialised practice or specialities with relatively small numbers of practitioners, whose expertise may be sought out by friends, family members, or colleagues. It has also been suggested that as the population ages, doctors in specialities such as oncology and palliative care may increasingly come across patients with whom they have existing social, collegial, or family relationships.

Professional boundaries also apply to roles not directly concerned with patient care, for example, in relation to medical and non-medical colleagues (‘corridor consultations’ and requests for prescriptions), and teaching and mentoring of students and junior colleagues. There is limited research on professional boundaries in relationships such as supervisor–trainee, faculty–student, and mentor–mentee. A potential for boundary violations arises from the power differential in the faculty–student (and supervisor–trainee) relationship which resides in the teacher’s (or supervisor’s) professional status and responsibility for evaluating the student’s (or trainee’s) skills, and the student’s vulnerability and dependence on the teacher for guidance and pass/fail grading.

### The connection between boundary challenges and burnout

Burnout has been described by pioneering researchers in the field as a psychological syndrome emerging as a prolonged response to chronic interpersonal stressors on the job.^12^ Early research identified key characteristics of burnout as emotional exhaustion, cynicism, reduced personal accomplishment, and depersonalisation.^13^ Consequences of burnout include erosion of compassion and reduced quality of patient care, reductions in work performance, increased risk of medical errors, prematurely leaving a career in medicine, and decreased life satisfaction.^14–16^

Boundary violations have been referred to as an ‘unrecognized risk for burnout’.^17^ The prevalence of burnout in doctors is uncertain, in part due to inconsistencies in research methodology and definition of the syndrome. In the Australian National Mental Health Survey of Doctors and Medical Students,^18^ rates of reported emotional exhaustion were high, especially in doctors under 30 years of age (47.5%). A recent review estimated a 21%–35% prevalence rate with significant increases since the COVID-19 pandemic.^19^ Higher levels of stress and burnout have been reported in younger doctors and in some specialities; gender associations are less consistent.^16^

There is a close connection between stress and burnout,^16^ and negotiating boundary challenges can be stressful, particularly in the absence of training and guidance.^20^ Most health professional codes of ethics and codes of conduct forbid some boundary violations explicitly, such as sexual relationships. However, there is little guidance in the literature and professional codes of practice regarding non-sexual boundary challenges. Many commonly encountered situations in professional settings may be complex and associated with ambiguity concerning boundary lines; the uncertainty in how to respond can exacerbate the associated stressfulness.^21^ Likewise, it can be difficult to refuse the request of a friend, family member, or colleague for a script or ‘corridor consultation’. Training has the potential to increase skills and confidence but is generally lacking at both undergraduate and post-graduate levels.

Peak psychology bodies appear to have given most consideration to guidance on professional boundaries. The Australian Psychological Society (APS) complements its ‘Code of Ethics’ with a series of 28 Ethical Guidelines, which apply the Code to issues encountered in everyday professional practice.^22^ However, a search of the Australian national associations of other health professions did not reveal any practical guidelines that can be applied to everyday professional practice to help professionals navigate boundary issues.

## Summary and recommendations

Effective management of non-sexual boundary challenges could contribute to a reduction in stress and burnout, help keep doctors in the profession, and increase patient safety. A greater understanding of the issues for Australian doctors and medical students in managing non-sexual boundary challenges would address a significant gap in the research literature. This may allow for the identification of educational targets and opportunities to provide the training and guidance that may lead to greater skill and confidence in managing boundary challenges.
